# Lipolytic agents for submental fat reduction: Review

**DOI:** 10.1111/srt.13601

**Published:** 2024-01-31

**Authors:** Soo Yeon Park, Soo‐Bin Kim, Jovian Wan, Fernando Felice, Kyu‐Ho Yi

**Affiliations:** ^1^ Made‐Young Plastic Surgery Clinic Seoul South Korea; ^2^ Division in Anatomy and Developmental Biology Department of Oral Biology Human Identification Research Institute BK21 FOUR Project Yonsei University College of Dentistry Seoul South Korea; ^3^ Asia Pacific Aesthetic Academy Seoul Hong Kong; ^4^ School of Medicine Universidad de Buenos Aires Buenos Aires Argentina; ^5^ Maylin Clinic (Apgujeong) Seoul South Korea

**Keywords:** aesthetic procedures, anatomical considerations, lipolytic agents, submental fat reduction

## Abstract

Result

The review delves into the realm of reducing submental fat, presenting a comprehensive analysis of various lipolytic agents used in plastic surgery and dermatology. The introduction establishes the context by defining the key indicators of a youthful neck and emphasizing the significant influence of fat in the aging process, particularly in the submental area. The usage of aminophylline involves subcutaneous injections, facilitating fat breakdown by increasing cyclic adenosine monophosphate and inhibiting adenosine receptors. Hypotonic pharmacologic lipo‐dissolution induces fat dissolution via injected compounds under pressure, while lipolytic lymphatic drainage employs hyaluronidase to reduce tissue viscosity, aiding fat circulation. Glycerophosphorylcholine containing choline alfoscerate claims to activate fat metabolism, whereas the utilization of phosphatidylcholine combined with deoxycholate lacks cosmetic approval due to safety concerns. Deoxycholic acid has FDA approval for submental fat reduction, yet its mechanisms remain incompletely understood. Understanding the complex anatomy and mechanisms of lipolytic agents is essential for safe and effective submental fat reduction, despite evolving practices and off‐label utilization. Clinical guidelines and references support this discussion, offering insights for safer applications.

## 1 INTRODUCTION

**FIGURE 1:**
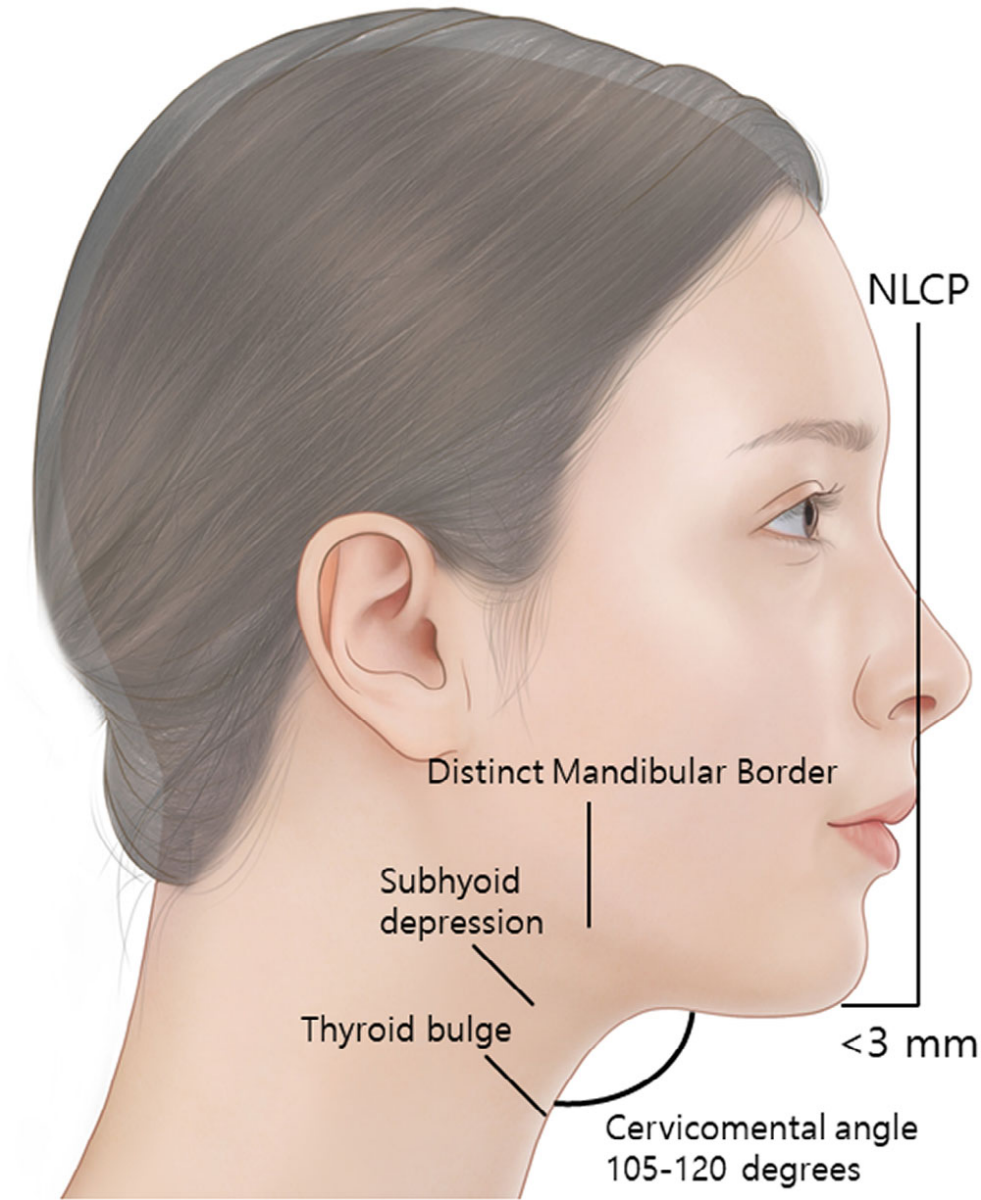
The figure demonstrates an illustrative representation of the cervicomental angle, portraying the characteristics of an ideal neck contour. It showcases a well‐defined mandibular border that forms an angle ranging between 105 and 120 degrees.

The term “youthful neck” generally refers to a well‐defined, creating a cervicomental angle of approximately 105–120 degrees. The most ideal scenario is when the sternocleidomastoid muscle and submental line form an angle of 90 degrees (Figure 1). Moreover, it is characterized by the existence of a subhyoid depression, prominence of the thyroid cartilage, and distinct visibility of the anterior border of the sternocleidomastoid muscle.^1^

From the most superficial layer to deeper layers, one can observe the skin, subcutaneous fat, and the platysma muscle, which, in turn lead to the deeper tissues such as the subplatysmal fat and submandibular gland. Beneath the platysma muscle, the cervical fascia envelops the suprahyoid muscles and submandibular gland, and above this fascia, certain cutaneous nerves and superficial veins pass through (Table 1). All tissues undergo aging processes and procedures, yet modifications in the fat tissue, which occupies the largest volume proportion, tend to exhibit a more pronounced effect.^2^ Hence, it becomes crucial to focus attention on this fat layer.

**TABLE 1:** Anatomical structure in the neck by layer dividing it to superficial, intermediate and deep.

Layer	Structures
Superficial	Skin Subcutaneous fat (pre‐platysmal fat)
Intermediate	Platysma muscle Interplatysmal fat
Deep	Soft tissue Subplatysmal fat (post‐platysmal fat) Digastric muscles Submandibular glands Skeletal support Mandible Hyoid bone

**FIGURE 2:**
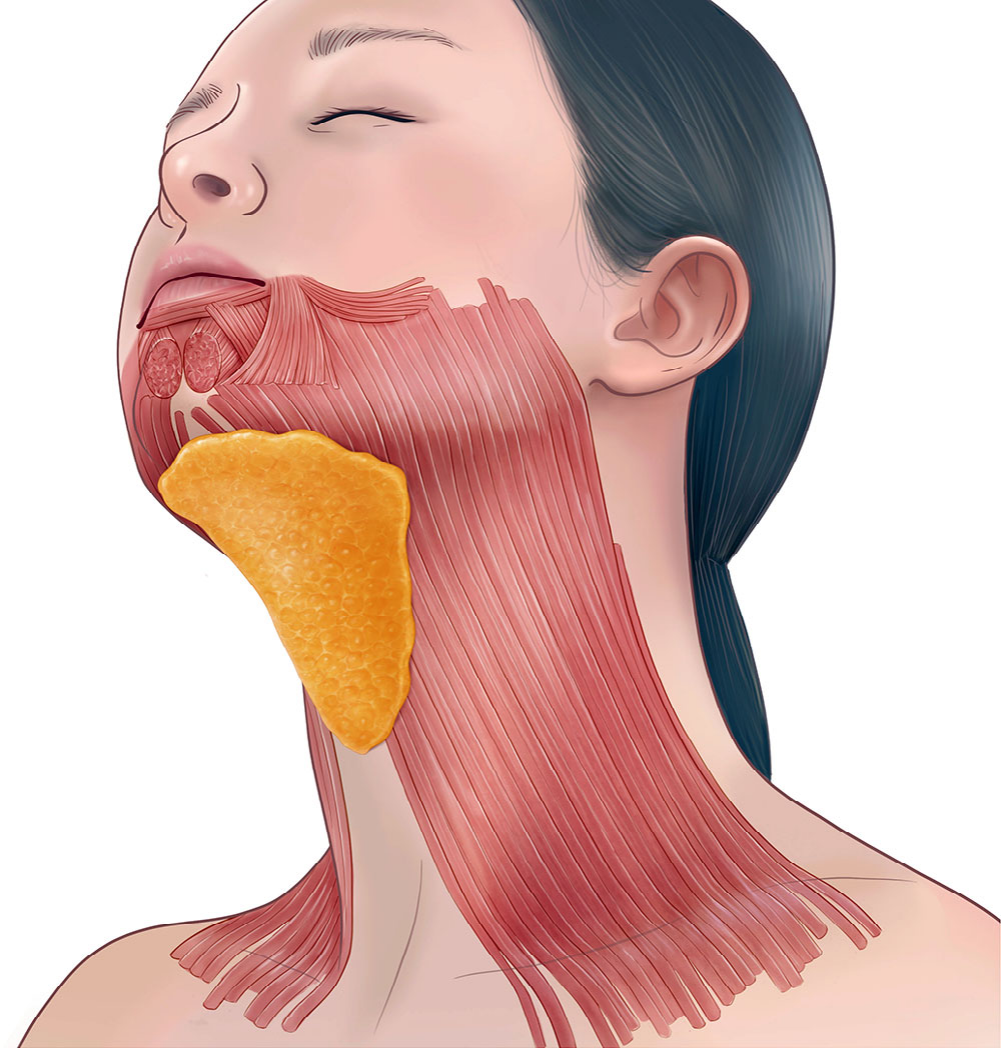
The figure illustrates the schematic image of the pre‐platysmal fat, which represents the primary targeted fatty layer for lipolytic agents.

As mentioned earlier, fat can be divided into preplatysmal fat and post‐platysmal fat, with the platysma muscle serving as the boundary, and both layers of fat contribute to the formation of a double chin in the submental area (Figure 2). Differentiating between these fats is clinically significant. This is because modifying the sub‐platysmal fat is challenging through non‐invasive methods and may potentially lead to deep tissue injury; thus, most procedures focus on the pre‐platysmal fat. The approach for deep fat often involves open surgical methods. The part that pinches upwards upon contraction of the platysma muscle is considered as the pre‐platysmal fat.^3^ Fat contouring can be broadly categorized into non‐surgical, minimally invasive, and surgical methods. Among the non‐surgical modalities used within plastic surgery and dermatology to reduce fat, one of the most frequently employed methods is the use of lipolytic agents.^4^ This review will encompass the agents frequently utilized for the reduction of submental fat.

## 2 AMINOPHYLLINE

Aminophylline, a soluble derivative of the xanthine theophylline (dimethylxanthine), possesses about 80% of the activity of theophylline. Originally used as a bronchodilator, it is administered intravenously by adding it to intravenous fluids when used for its intended purpose. However, for localized injection therapy, it is administered in the form of subcutaneous injections.^5^ Although there is no clear evidence regarding its safety and efficacy mechanisms, reports from various studies suggest its effectiveness, leading to its widespread use in many medical institutions for this purpose.^6^ When combined with free hyaluronic acid, which has properties for retaining water molecules, it regulates the rate of aminophylline elimination, thereby prolonging the drug's effect at the injection site and subsequently increasing the localized fat‐reducing effect. The promotion of fat breakdown by xanthine derivatives is explained through three pathways: Firstly, by inhibiting phosphodiesterase type IIIB, thereby increasing intracellular cyclic adenosine monophosphate and inducing protein kinase activation.^7^ Secondly, by inhibiting adenosine receptors, which promotes fat breakdown. Thirdly, aminophylline acts as a beta‐2 adrenergic agonist, stimulating fat breakdown.^5^

## 3 HYPOTONIC PHARMACOLOGIC LIPO‐DISSOLUTION

Subsequently, an overview of hypotonic pharmacologic lipo‐dissolution (HPL), frequently utilized in lipo‐dissolution procedures, will be provided. This technique entails the injection of solutions containing fat‐storing compounds and agents that facilitate fat breakdown into the subcutaneous adipose tissue. The injected substances induce adipocytes to swell and segregate under hydrostatic pressure, thereby fostering lipid dissolution through their collective interactions.^8^ The dissolved fat is absorbed through the lymphatic vessels and eliminated from the body as waste through urine. Utilization of ultrasound or low‐level laser therapy enhances the efficacy of fat dissolution. Low‐level laser therapy, in particular, has been employed for fat breakdown upon the discovery that fat dissolution occurs at specific frequencies. This approach, initially proposed by Dr Hoefflin, has undergone modifications and is currently utilized in various clinics for clinical applications (Table 2).^8^

**TABLE 2:** Hoefflin's HPL solution is hypotonic pharmacologic lipo‐dissolution (HPL) technique proposed by Dr Hoefflin.

Ingredient	Function
400 mL of N/S solution	
600 mL Sterile water	
0.25 mL 1:1000 epinephrine	Stimulate catecholamine Intracellular lipolysis and subsequent cellular osmotic gradient
1 mL dehydrated alcohol	Inhibits cellular lipid interface
2 mL potassium chloride	Assists in elevating sodium potassium osmotic gradient
0.5 mL verapamil	Inhibits the cell wall Ca2 = dependent fluid transport
0.5 mL triamcinolone 40 mg/mL	Assists in secondary resolution of edema

*Note*: This method involves the injection of specific solutions containing compounds aimed at inducing fat breakdown within the subcutaneous adipose tissue.

## 4 LIPOLYTIC LYMPHATIC DRAINAGE

Lipolytic lymphatic drainage primarily involves hyaluronidase, which, when injected into the body, breaks down hyaluronic acid, reducing tissue viscosity. This decrease in viscosity facilitates fat circulation through the lymph nodes. Essentially, it promotes local lymphatic circulation, aiding in the improvement of cellulite and edema. Hyaluronidase possesses the property of hydrolyzing substances within the skin tissue, aiding in their dissolution. By utilizing its action on connective tissue dissolution, injecting it subcutaneously leads to loosening of abdominal connective tissue, reducing tissue edema. Consequently, this assists in enhancing blood and lymphatic circulation, facilitating the breakdown and metabolism of abdominal fat cells.^9^

## 5 GLYCEROLPHOSPHORYLCHOLINE

Furthermore, choline alfoscerate contains glycerophosphorylcholine, a precursor to choline, a major component of neuronal cell membrane lipids. It serves a role in forming cellular membranes and plays a role in neurotransmission as a precursor to acetylcholine.^10^ Choline is purported to activate fat metabolism when administered in significant amounts; however, its approval by the FDA is currently limited to intravenous injection or oral therapy. The use of choline for subcutaneous injections to destroy fat is not yet an approved method and falls outside the scope of authorization.

## 6 PHOSPHATIDYLCHOLINE

Understanding of phosphatidylcholine (PPC) is necessary, which differs slightly from the previously mentioned glycerolphosphorylcholine. Although the authors are currently not utilizing PPC itself, have experience with deoxycholate. Hence, acquiring an understanding of PPC is pertinent. Originally derived from soybean oil, PPC is known to be involved in triglyceride metabolism. It has been utilized to inhibit fat accumulation, addressing conditions such as cardiovascular diseases and fatty liver. Deoxycholate acid (DCA) has served a detergent role in PPC formulations. Consequently, PPC combined with DCA has been widely used as a lipolytic agent. Initially introduced as an intravenous agent known as lipostabil, it has been off‐label employed in some instances for obesity treatment.^11^ However, due to insufficient evidence regarding the safety of the PPC combination with DCA compound for cosmetic fat reduction, its usage has been prohibited. Subsequently, the focus shifted away from PPC to DCA, leading to several research endeavors.^7^.

## 7 DEOXYCHOLIC ACID

DCA functions as a detergent, disrupting membrane integrity and causing cell collapse, as previously mentioned.^12^ When administered alone via injection, fat necrosis was observed, targeting fat more effectively than other tissues due to its differential affinity for albumin. The first FDA‐approved product in 2015 was Kybella, which was imported and distributed in South Korea as “Belkyra.” However, its relatively high cost initially limited its widespread popularity. Last year, Daewoong introduced a similar drug called “V‐olet,” containing the same active ingredient. Presently, it is widely used due to increased affordability. Although its approval is limited to submental fat, the potential for off‐label use in other areas exists.^13^ DCA, commonly used for moderate to severe submental fat indications, still lacks a fully clarified mechanism. Recent in vitro research presents conflicting results regarding DCA's effect. Some studies suggest non‐specific necrosis induced by DCA, while others propose fat‐specific lipolysis by PPC. These contradictory findings underscore the need for additional research to achieve a comprehensive understanding.^14^

Some of the adverse effects associated with DCA injections include initial edema resulting in dysphagia, numbness, and induration, which usually resolve naturally over time. One of the most concerning side effects is the development of skin ulcers if the injection is superficial; in such cases, early care with medications like prednisolone and antibiotics might help, but severe cases might require surgery. In men, there is a possibility of alopecia if injected into areas with facial hair, yet this typically resolves naturally within 3 months, and intradermal prednisolone injections may aid recovery. Improper sterile procedures during injections could lead to infectious complications such as cellulitis or abscess formation. Furthermore, there is a potential risk of partial injury to the marginal mandibular nerve.^12^ Certainly, most adverse effects, including nerve injuries, tend to resolve naturally within a period ranging from three weeks to 6 months without specific intervention.^15^ Certainly, due attention is warranted for the marginal mandibular nerve in the submental region, given its path. Arising from the caudal edge of the parotid gland or below the angle, it courses anteriorly along the mandible's inferior border, turning upward at the mid‐mandibular body, known as the antegonial notch, traversing through the deep cervical fascia. Injury in this course may lead to weakened functions of the depressor anguli oris and depressor labii inferioris, resulting in asymmetrical smiles or asymmetry in lip movement.^16^ Similarly, the cervical branch of the facial nerve follows a comparable path, innervating the platysma muscle, affecting lip depression but not lip eversion. Such nerve injuries are attributed to demyelination or inflammation caused by deoxycholate, and evidence suggests their dose‐dependent occurrence. Additionally, inflammation in the platysma might induce neuropraxia‐like symptoms, where confirming lip pouting can distinguish nerve involvement; administering botulinum toxin to the non‐paralyzed depressor anguli oris can mitigate symptoms. Primarily, anti‐inflammatory agents such as prednisolone, Ibuprofen, or intravenous administration of vitamin B complex are utilized to alleviate inflammation. Caution is advised, especially in patients with thin or atrophic platysma or those with prior neck procedures, as facial contour surgeries or suction procedures may cause scarring and contractures, altering nerve pathways. Recovery duration can range from 1 week to approximately 10 months, with longer recovery periods often required for the marginal mandibular nerve compared to the cervical nerve. Hence, consideration of anti‐inflammatory agents like prednisolone is recommended.^15^

Off‐label use of deoxycholic acid injections has been reported in clinical cases for body contouring and lipomas, beyond the approved submental fat indication, and has been attempted in various clinics.^17^ For jowl fat treatment, injections are often administered in volumes less than 0.5 cc or diluted in distilled water (not saline) to a concentration of about 0.5%, approximately half the standard 1%, yielding promising outcomes.^18^ Posterior and anterior axillary lines have also been targeted for treatments, with reported cases showing effective maintenance lasting up to 3 to 9 months post‐treatment.^19^

## 8 STEROID

Local injections of cortisol are known to elevate circulating free fatty acids. This correlation has been traditionally associated with the ability of glucocorticoids to promote lipolysis. The study of Xu et al.,^20^ conducted using rat primary adipocytes, demonstrates that glucocorticoids directly induce lipolysis in these cells in a manner that is both dose‐ and time‐dependent. This effect was reduced by RU486, a glucocorticoid antagonist. Dexamethasone, a glucocorticoid, decreased cyclic‐nucleotide phosphodiesterase 3B levels, leading to increased cyclic adenosine monophosphate and activated PKA, initiating lipolysis. RU486 or H89, a PKA inhibitor, reversed these effects. Dexamethasone also increased hormone‐sensitive lipase and adipose triglyceride lipase levels, associated with enhanced lipolysis, counteracted by RU486 or actinomycin D. Despite these changes, hormone‐sensitive lipase did not relocate to lipid droplets in mature adipocytes. In vivo experiments in dexamethasone‐treated rats showed elevated FFAs in correlation with increased lipase activity, affirming the link between glucocorticoids and systemic FFA elevation. In certain cases, local injections of steroids can lead to localized lipoatrophy.^21^ Despite this risk, many clinics still frequently combine steroids like dexamethasone and triamcinolone with other agents to achieve lipolytic effects.

## 9 DISCUSSION

A double chin, also known as submental fat, has a negative effect on the appearance of the face aesthetically, and surgical and non‐surgical treatments have been used for treatment.^22^ Surgical treatments, including liposuction and platysmalplasty, have been employed. Non‐surgical methods involve the use of energy‐based devices such as high‐frequency and high‐intensity focused ultrasound equipment, or the induction of adipocytolysis through various injection therapies.^23^

Within the realm of injection therapies, DCA injection therapy is recognized for inducing an inflammatory response. This response involves the mobilization of macrophages, promotion of fibroblast formation, and stimulation of collagen formation when injected into subcutaneous fat. The DCA formulation was initially introduced by Kythera Biopharmaceuticals in the United States for the purpose of reducing submental fat. Following approval by the Food and Drug Administration, DCA was released in South Korea in 2021 by Daewoong Pharmaceutical.^3^

Cosmetic and dermatological applications of lipolytic agents have broadened significantly, even capable of replacing liposuction in appropriately selected patient populations. With proper patient selection, these agents can yield outcomes comparable to surgery but with minimal downtime, allowing for a wide range of procedures. They can be particularly beneficial for patients reluctant to undergo secondary liposuction procedures or those wary of more burdensome secondary facial contouring. Despite deoxycholic acid's current approval limited to pre‐platysmal fat, accumulating off‐label cases demonstrate its expanding utility, raising hopes for stabilized protocols across various applications.

In conclusion, this comprehensive review underscores the evolving landscape of lipolytic agents for submental fat reduction, emphasizing the need for further research into their mechanisms and safety profiles. Understanding the anatomical intricacies and potential adverse effects associated with these agents is crucial for practitioners aiming to achieve optimal outcomes while ensuring patient safety in cosmetic and dermatological procedures.

## AUTHOR CONTRIBUTIONS

All authors have reviewed and approved the article for submission. Conceptualization, Kyu‐Ho Yi, Soo Yeon Park. Writing—Original Draft Preparation, Kyu‐Ho Yi, Soo Yeon Park, Jovian Wan. Writing—Review & Editing, Kyu‐Ho Yi, Soo Yeon Park, Jovian Wan. Visualization, Kyu‐Ho Yi, Soo Yeon Park, Fernando Felice. Supervision, Kyu‐Ho Yi, Soo Yeon Park, Soo‐Bin Kim, Jovian Wan

## CONFLICT OF INTEREST STATEMENT

I acknowledge that I have considered the conflict of interest statement included in the “Author Guidelines.” I hereby certify that, to the best of my knowledge, that no aspect of my current personal or professional situation might reasonably be expected to significantly affect my views on the subject I am presenting.

## Data Availability

The data that support the findings of this study are available on request from the corresponding author. The data are not publicly available due to privacy or ethical restrictions.

## References

[1] Ellenbogen R , Karlin JV . Visual criteria for success in restoring the youthful neck. Plast Reconstr Surg. 1980;66(6):826‐837. 7443846 10.1097/00006534-198012000-00003

[2] Weinstein AL , Nahai F . A layered approach to neck lift. Plast Aesthet Res. 2021;8:11.

[3] Shamban AT . Noninvasive Submental Fat Compartment Treatment. Plast Reconstr Surg Glob Open. 2016;4(12 Suppl Anatomy and Safety in Cosmetic Medicine: Cosmetic Bootcamp):e1155. 28018773 10.1097/GOX.0000000000001155 PMC5172481

[4] Pérez P , Hohman MH . Neck Rejuvenation. In: StatPearls. StatPearls Publishing.; 2023. 32965900

[5] Miyoshi Y , Uchida K , Takeda‐Hara E , Nagai K , Okuda H . The mechanism of the lipolytic action of theophylline in fat cells. Pharmacol Biochem Behav. 1981;14(5):701‐706. 7243846 10.1016/0091-3057(81)90134-9

[6] Abdi Dezfouli R , Hosseinpour A , Qorbani M , Daneshzad E . The efficacy of topical aminophylline in local fat reduction: a systematic review. Front Endocrinol. 2023;14:1087614. 10.3389/fendo.2023.1087614 PMC9978326 36875487

[7] Caruso MK , Roberts AT , Bissoon L , Self KS , Guillot TS , Greenway FL . An evaluation of mesotherapy solutions for inducing lipolysis and treating cellulite. J Plast Reconstr Aesthet Surg. 2008;61(11):1321‐1324. 17954040 10.1016/j.bjps.2007.03.039

[8] Hoefflin SM . Lipoplasty with hypotonic pharmacologic lipo‐dissolution. Aesthet Surg J. 2002;22(6):1321‐1324. 10.1067/maj.2002.129980 19332017

[9] Jung H . Hyaluronidase: an overview of its properties, applications, and side effects. Arch Plast Surg. 2020;47(4):573‐576. 10.5999/aps.2020.00752 PMC7398804 32718106

[10] Kim GW , Chung SH . The beneficial effect of glycerophosphocholine to local fat accumulation: a comparative study with phosphatidylcholine and aminophylline. Korean J Physiol Pharmacol. 2021;25(4):333‐339. 34187950 10.4196/kjpp.2021.25.4.333 PMC8255124

[11] Noh Y , Heo CY . The effect of phosphatidylcholine and deoxycholate compound injections to the localized adipose tissue: an experimental study with a murine model. Arch Plast Surg. 2012;39(5):333‐339. 23094238 10.5999/aps.2012.39.5.452 PMC3474400

[12] Jalian HR , Fitzgerald R , Bowen B , Gamio S . Submental fat reduction using sequential treatment approach with cryolipolysis and ATX‐101. J Cosmet Dermatol. 2022;21(6):2437‐2444. 35278262 10.1111/jocd.14909 PMC9325515

[13] Kim H‐M , Ree Y‐S , Park M‐S , Kim J‐S , Ahn J‐H , Yi K‐H . Clinical guideline: deoxycholic acid injection for submental fat reduction. The Aesthetics. 2022;3:2437‐2444.

[14] Muskat A , Pirtle M , Kost Y , McLellan BN , Shinoda K . The role of fat reducing agents on adipocyte death and adipose tissue inflammation. Frontiers in Endocrinology.7‐11, 2022;13. 10.3389/fendo.2022.841889 PMC8988282 35399925

[15] Blandford AD , Ansari W , Young JM , et al. Deoxycholic acid and the marginal mandibular nerve: a Cadaver Study. Aesthetic Plast Surg. 2018;42(5):1394‐1398. 29869228 10.1007/s00266-018-1164-4

[16] Yi KH , Lee JH , Hu HW , et al. Novel anatomical proposal for botulinum neurotoxin injection targeting depressor anguli oris for treating drooping mouth corner. Anat Cell Biol. 2023;56(2):1394‐1398. 10.5115/acb.22.258 PMC10319486 36808109

[17] Sutaria A , Kapoor A , Sharma YK , Gupta A . Deoxycholic acid injection in the management of difficult‐to‐remove subcutaneous lipomas. Dermatol Surg. 2022;48(3):161‐165. 10.1097/DSS.0000000000003343 35125438

[18] Shridharani SM . Improvement in jowl fat following ATX‐101 treatment: results from a single‐site study. Plast Reconstr Surg. 2020;145(4):367‐368. 10.1097/PRS.0000000000006680 PMC7099849 32221205

[19] Jegasothy SM . Deoxycholic Acid Injections for bra‐line lipolysis. Dermatol Surg. 2018;44(5):929‐935. 10.1097/DSS.0000000000001311 PMC5943074 29016540

[20] Xu C , He J , Jiang H , et al. Direct effect of glucocorticoids on lipolysis in adipocytes. Mol Endocrinol. 2009;23(8):757‐760. 10.1210/me.2008-0464 PMC5419195 19443609

[21] Kim MJ , Park HJ , Oh SM , Yi KH . Polynucleotide injection treatment for iatrogenic fat atrophy in two patients: potential for safe volumization in aesthetic medicine. Skin Res Technol. 2023;29(8):1161. 10.1111/srt.13439 PMC10423761 37632185

[22] Ascher B , Hoffmann K , Walker P , Lippert S , Wollina U , Havlickova B . Efficacy, patient‐reported outcomes and safety profile of ATX‐101 (deoxycholic acid), an injectable drug for the reduction of unwanted submental fat: results from a phase III, randomized, placebo‐controlled study. J Eur Acad Dermatol Venereol. 2014;28(12):1707‐1715. 24605812 10.1111/jdv.12377 PMC4263247

[23] Humphrey S , Sykes J , Kantor J , et al. ATX‐101 for reduction of submental fat: a phase III randomized controlled trial. J Am Acad Dermatol. 2016;75(4):788‐797.e787. 27430612 10.1016/j.jaad.2016.04.028

